# Suicide in cancer patients in South East England from 1996 to 2005: a population-based study

**DOI:** 10.1038/sj.bjc.6605110

**Published:** 2009-05-26

**Authors:** D Robinson, C Renshaw, C Okello, H Møller, E A Davies

**Affiliations:** 1 Division of Cancer Studies, King's College London, Thames Cancer Registry, 1st Floor Capital House, 42 Weston Street, London SE1 3QD, UK

**Keywords:** suicide, standardised mortality ratio, fatality, deprivation

## Abstract

BACKGROUND: Studies from around the world have shown that suicide risk is increased in cancer patients, but no previous detailed analysis has been carried out in England.

METHODS: We calculated standardised mortality ratios (SMRs) for suicide in 206 129 men and 211 443 women diagnosed with cancer in South East England between 1996 and 2005, relative to suicide rates in the general population.

RESULTS: We found a significantly increased risk of suicide in men (SMR 1.45, 95% confidence interval (CI) 1.20–1.73) and a moderately increased risk in women (SMR 1.19, 95% CI 0.88–1.57). In both sexes, relative risk of suicide was greatest in the first year after cancer diagnosis (SMR for men 2.42, 95% CI 1.84–3.13; SMR for women 1.44, 95% CI 0.82–2.33), and was also greater in individuals diagnosed with types of cancer with high fatality (SMR for men 2.67, 95% CI 1.71–3.97; SMR for women 2.17, 95% CI 0.80–4.73).

CONCLUSION: There is a critical period immediately after the diagnosis of cancer during which the excess risk of suicide is particularly high. Carers need to be aware of the importance of attending to both the physical and emotional needs of cancer patients and cancer survivors.

Patients diagnosed with life-threatening conditions such as cancer experience painful emotional reactions, which can lead to suicidal thoughts. The risk of suicide in cancer patients has been reported as elevated relative to that of the general population in several countries: Italy (Crocetti *et al*, 1998; Miccinesi *et al*, 2004), Switzerland (Chatton-Reith *et al*, 1990; Levi *et al*, 1991), Denmark (Storm *et al*, 1992; Yousaf *et al*, 2005; Christensen *et al*, 2006; Schairer *et al*, 2006), Norway (Hem *et al*, 2004; Schairer *et al*, 2006), Sweden (Allebeck *et al*, 1989; Allebeck and Bolund, 1991; Björkenstam *et al*, 2005; Schairer *et al*, 2006; Björkholm *et al*, 2007), Finland (Louhivuori and Hakama, 1979; Schairer *et al*, 2006), Estonia (Innos *et al*, 2003), Australia (Dormer *et al*, 2008), Japan (Tanaka *et al*, 1999), Scotland (Camidge *et al*, 2007) and the United States of America (Fox *et al*, 1982; Llorente *et al*, 2005; Kendal, 2007; Miller *et al*, 2008; Misono *et al*, 2008). The majority of studies report a higher relative risk in men than in women. The risk has been shown to be greatest in the first months after diagnosis (Crocetti *et al*, 1998; Tanaka *et al*, 1999; Innos *et al*, 2003; Hem *et al*, 2004; Llorente *et al*, 2005; Yousaf *et al*, 2005; Dormer *et al*, 2008), and to increase with the severity of the disease (Louhivuori and Hakama, 1979; Storm *et al*, 1992; Tanaka *et al*, 1999; Miccinesi *et al*, 2004; Björkenstam *et al*, 2005; Yousaf *et al*, 2005; Kendal, 2007; Dormer *et al*, 2008).

A series of recent initiatives in cancer care in England have sought to improve the provision and quality of supportive and palliative care services (National Institute for Clinical Excellence, 2004), and to improve the quality of life of cancer survivors (Department of Health, 2007). In the year following the diagnosis of cancer, around 1 in 10 patients are reported to experience symptoms of either depression or anxiety severe enough to require intervention by specialist psychological or psychiatric services (National Institute for Clinical Excellence, 2004). To our knowledge no previous detailed analysis of suicide risk in individuals with cancer has been carried out in England, although a recent study, covering the period 1981 to 1995, has reported on hospital admissions and deaths relating to deliberate self-harm in cancer patients in Scotland (Camidge *et al*, 2007). In this study, we aim to provide new information relating to suicide risk in patients diagnosed with cancer in South East England during the more recent time period of 1996 to 2005.

## Methods

The records of all patients diagnosed with an invasive cancer between 1996 and 2005 were extracted from the database of the Thames Cancer Registry. This is a population-based registry, covering around 12 million people residing in an area of South East England comprising London, Kent, Surrey and Sussex. We excluded patients registered only from a death certificate or for whom the recorded date of diagnosis was the same as the date of death (9.5% of all cases), and those with a date of death but no recorded cause of death (0.54% of all deaths). We also excluded patients aged less than 15 years at cancer diagnosis, as suicide rates in the general population are not available for this age group. Cases of suicide were identified either from the ICD cause of death code (where present) or from appropriate text fields in the death certificate. The relevant ICD-9 codes were E950-E959 and E980-E989 (excluding E988.8), with corresponding ICD-10 codes X60-X84 and Y10-Y34 (but excluding Y33.9 where a coroner's verdict was pending). These were chosen to be consistent with those used by National Statistics (Brock *et al*, 2006). Keywords searched for in the text fields were ‘suicide’, ‘killed him/herself’, ‘own life’ and ‘overdose’. Of the 166 suicides identified, 8 (4.8%) were found by text analysis alone.

Standardised mortality ratios (SMRs) were calculated by dividing the observed numbers of suicides by the numbers expected, derived from age/sex/calendar period-specific suicide rates for the London and South East Coast Strategic Health Authorities provided by the Office for National Statistics. Time at risk was calculated from cancer diagnosis to death or end of the study period (31 December 2005). For individuals with more than one cancer diagnosis, the date of the first cancer was taken as the start of the at-risk period. SMRs were calculated separately for men and women, and were examined in relation to a number of factors: time since cancer diagnosis, age at diagnosis, stage of disease (1=localised; 2=extension beyond the organ of origin; 3=local lymph node involvement; 4=metastases), fatality of the cancer type (based on relative survival figures, with those cancer types with a 5-year survival of less than 10% being classified as having ‘high’ fatality), calendar period of diagnosis and deprivation (measured in quintiles using the income domain of the Indices of Multiple Deprivation (IMD) 2004, assigned on the basis of place of residence at the time of diagnosis (Neighbourhood Renewal Unit, 2004)). A Cox proportional hazards regression model was fitted using all of these factors as covariates, in an attempt to estimate their independent and joint effects on the risk of committing suicide after being diagnosed with cancer.

## Results

A total of 206 129 male and 211 443 female cancer patients with a combined follow-up of 1.1 million person-years were included in the analysis. Among these, we observed 166 suicides (117 in men and 49 in women). The mean age at diagnosis of first cancer in those committing suicide was 67.9 years in men and 63.4 years in women. Mean age at suicide was 69.9 years in men and 65.9 years in women. Table 1 shows the SMRs and 95% confidence intervals (CIs) for suicide by sex and time since diagnosis. Overall, there was a significantly increased risk of suicide in men (SMR 1.45; 95% CI 1.20–1.73). In women, the SMR was lower and did not reach statistical significance (SMR 1.19, 95% CI 0.88–1.57). In both sexes there was a downward trend in relative risk of suicide with increasing time since diagnosis: SMRs at <1, 1–5 and >5 years after diagnosis were 2.42 (1.84–3.13), 1.04 (0.76–1.39) and 1.01 (0.54–1.73) for men and 1.44 (0.82–2.33), 1.10 (0.71–1.63) and 1.08 (0.47–2.14) for women.

Table 2 shows the mutually adjusted hazard ratios from the Cox regression analysis. In both men and women, the risk of suicide was highest in the first year after cancer diagnosis. Overall, there was a suggestion of increasing risk of suicide with increasing age. The more fatal types of cancer carried the highest risk of suicide in both men and women, but a strong effect of advanced stage of disease was evident only in women. There were no significant trends in suicide risk over the period studied. There was a tendency for those from more deprived areas (higher IMD 2004 quintiles) to be at greater risk than those from more affluent areas: suicide risk was highest in the socio-economically deprived groups of women.

## Discussion

We have found an excess of suicides in patients diagnosed with cancer in South East England, which is most pronounced in the first year following diagnosis and higher in men than in women. These results are generally consistent with previous findings. In the majority of studies that have compared suicide risks between the sexes, men were found to have a higher relative risk than women (Fox *et al*, 1982; Allebeck *et al*, 1989; Levi *et al*, 1991; Innos *et al*, 2003; Hem *et al*, 2004; Miccinesi *et al*, 2004; Yousaf *et al*, 2005; Dormer *et al*, 2008; Misono *et al*, 2008).

In both men and women we found the relative risk of suicide to be greatest in the first year after diagnosis. This is also consistent with many of the previous studies, which found a maximum risk within the first few months (Crocetti *et al*, 1998; Tanaka *et al*, 1999; Innos *et al*, 2003; Hem *et al*, 2004; Llorente *et al*, 2005; Yousaf *et al*, 2005; Dormer *et al*, 2008) or the first 1–2 years (Allebeck *et al*, 1989; Chatton-Reith *et al*, 1990; Levi *et al*, 1991; Storm *et al*, 1992; Miccinesi *et al*, 2004; Björkholm *et al*, 2007). Dormer *et al* (2008) found a peak risk in the first 3 months, with a second period of increased risk 12–14 months after diagnosis, which they suggested might be a response to cancer recurrence or treatment failure. An international study of women with breast cancer (Schairer *et al*, 2006) found an elevated risk throughout the follow-up period, including more than 25 years after diagnosis.

Some investigators (Chatton-Reith *et al*, 1990; Miccinesi *et al*, 2004; Schairer *et al*, 2006) have found an increase in relative risk with increasing age. Others (Fox *et al*, 1982; Allebeck *et al*, 1989; Levi *et al*, 1991; Björkenstam *et al*, 2005; Yousaf *et al*, 2005; Dormer *et al*, 2008) found no such relationship, whereas Innos *et al* (2003) found the highest risk in men aged 15–49, with a tendency for the risk to decline in older age groups. In our study, there was a suggestion of increasing suicide risk with increasing age.

Suicide risk has been shown to be associated with stage of disease, with higher risks in patients with non-localised disease (Louhivuori and Hakama, 1979; Storm *et al*, 1992; Yousaf *et al*, 2005) or distant metastases (Tanaka *et al*, 1999; Kendal, 2007). In a case–control study of Americans aged 65 or more (Miller *et al*, 2008), the only medical condition found to be associated with suicide was cancer, and metastases were significantly more common in cancer patients who committed suicide than in those who did not. In our study, the risk of suicide was significantly higher in women with later stage disease, but not in men. We found a significantly raised SMR in men with unknown stage (1.78, 95% CI 1.31–2.37). However, this group of patients were on average older and had shorter survival than the staged patients, and the relationship became non-significant after adjusting for these factors in the full model.

Suicide risk has also been shown to be higher in patients diagnosed with cancers that have a poor prognosis (Storm *et al*, 1992; Yousaf *et al*, 2005; Dormer *et al*, 2008) or a low survival rate (Miccinesi *et al*, 2004; Björkenstam *et al*, 2005). Misono *et al* (2008) found the risk to be highest for lung, stomach or head and neck cancers, whereas Innos *et al* (2003) found the greatest risks in men with cancers of the oesophagus or pancreas. We found significantly higher risks associated with cancers at sites with high fatality rates (5-year relative survival of less than 10%) when compared with those at other sites, with SMRs of 2.42 (1.84–3.13) for men and 1.44 (0.82–2.33) for women.

There is some evidence from other studies (Hem *et al*, 2004; Miccinesi *et al*, 2004; Björkenstam *et al*, 2005) that the relative risk of suicide in cancer patients is decreasing in more recent years. This may be due to new treatments, increased survival and a better public awareness that cancer is not necessarily a fatal condition – all of which should improve a patient's reaction to a diagnosis of cancer. However, Fox *et al* (1982) found no significant trend with decade of diagnosis, and two Danish studies (Storm *et al*, 1992; Yousaf *et al*, 2005) found increasing risks over time. After adjusting for confounding factors, we found no significant trend over time in the population of South East England.

Chatton-Reith *et al* (1990) found suicide relative risk to be lower in the upper socio-economic classes. There is a suggestion of this in our data, with lower risks in the two most affluent quintiles, although the differences did not reach statistical significance and were attenuated after adjustment for other factors, including disease stage.

The results of our and other studies indicate that there is a critical period immediately after diagnosis during which the excess risk of suicide in cancer patients is particularly high. Patients who survive this initial period are much less likely to commit suicide, although the risk can remain elevated for many years. The risk of suicide is especially high in those diagnosed with cancers such as lung, pancreas or oesophagus, where the prognosis is poor and the symptoms painful or likely to interfere with vital functions such as breathing and eating. The SMRs for these cancers in the first year after diagnosis were 4.60 (2.51–7.72), 6.79 (1.40–19.83) and 6.52 (2.12–15.22) respectively.

Studies (Henriksson *et al*, 1995; Akechi *et al*, 2001, 2002a, 2002b; Filiberti *et al*, 2001; Smith *et al*, 2004; Walker *et al*, 2008) have consistently shown that substantial pain, major depression, loss of autonomy and independence, emotional distress and impaired physical functioning are the clinical factors most commonly associated with suicidal thoughts in cancer patients. One recent survey of nearly 3000 cancer patients attending a regional cancer centre in Scotland found that almost 8% reported having thoughts of being better off dead or of hurting themselves in some way for at least several days in the previous 2 weeks (Walker *et al*, 2008). As pointed out, not all of these patients will attempt to harm themselves, but acknowledgment of emotional distress should be a core aspect of modern cancer care, together with careful control of symptoms such as pain. As stressed by Maliski *et al* (2003), awareness is important in attending to both the physical and emotional needs of cancer patients and cancer survivors.

## Figures and Tables

**Table 1:** Standardised mortality ratios for suicide in cancer patients, by sex and time since diagnosis

	**Years since diagnosis**			
	**<1**	**1–5**	**>5**	**Total**
Suicides	58	46	13	117
Males				
SMR	2.42	1.04	1.01	1.45
95% CI	1.84–3.13	0.76–1.39	0.54–1.73	1.20–1.73
				
Suicides	16	25	8	49
Females				
SMR	1.44	1.10	1.08	1.19
95% CI	0.82–2.33	0.71–1.63	0.47–2.14	0.88–1.57
				
Suicides	74	71	21	166
All				
SMR	2.11	1.06	1.04	1.36
95% CI	1.65–2.65	0.83–1.34	0.64–1.59	1.16–1.58

SMR=standardised mortality ratio; CI=confidence interval.

**Table 2:** Adjusted hazard ratios and 95% confidence intervals for suicide in cancer patients in South East England 1996–2005

**Factor**	**Males**	**Females**	**All**
*Time since diagnosis (years)*			
0–1	2.09 (1.39–3.15)	1.14 (0.59–2.22)	1.78 (1.26–2.50)
>1	1.00	1.00	1.00
			
*Age at diagnosis (years)*			
15–60	1.00	1.00	1.00
61–75	0.78 (0.24–2.59)	2.88 (0.46–18.04)	1.29 (0.47–3.54)
>75	1.24 (0.24–6.44)	1.37 (0.10–18.66)	1.57 (0.40–6.17)
			
Stage			
1–2	1.00	1.00	1.00
3–4	0.79 (0.43–1.44)	2.41 (1.25–4.64)	1.16 (0.76–1.76)
N/K	1.38 (0.94–2.05)	1.10 (0.53–2.29)	1.31 (0.93–1.85)
			
Fatality of cancer			
Low	1.00	1.00	1.00
High	1.76 (1.10–2.81)	1.57 (0.65–3.81)	1.87 (1.24–2.82)
			
Period of diagnosis			
1996–1999	1.00	1.00	1.00
2000–2002	0.98 (0.64–1.49)	0.96 (0.50–1.83)	1.00 (0.70–1.42)
2003–2005	1.10 (0.66–1.85)	1.07 (0.44–2.57)	1.12 (0.72–1.75)
			
IMD 2004 quintile			
1–2	1.00	1.00	1.00
3	1.07 (0.65–1.75)	1.76 (0.84–3.70)	1.24 (0.82–1.87)
4–5	1.06 (0.71–1.60)	1.45 (0.75–2.82)	1.17 (0.83–1.65)
